# Inclusion of Oat in Feeding Can Increase the Potential Probiotic Bifidobacteria in Sow Milk

**DOI:** 10.3390/ani5030375

**Published:** 2015-07-22

**Authors:** Rabin Gyawali, Radiah C. Minor, Barry Donovan, Salam A. Ibrahim

**Affiliations:** 1 Food Microbiology and Biotechnology Laboratory, 173 Carver Hall, North Carolina Agricultural and Technical State University, Greensboro, NC 27411, USA; E-Mail: rgyawali@aggies.ncat.edu; 2 Department of Animal Sciences, 107h Webb Hall, North Carolina Agricultural and Technical State University, Greensboro, NC 27411, USA; E-Mails: rcminor@ncat.edu (R.C.M.); bcdonova@gmail.com (B.D.)

**Keywords:** oat, sow milk, probiotic, bifidobacteria, isolation, characterization

## Abstract

**Simple Summary:**

In this study we isolated and characterized potential probiotic bifidobacteria from sow milk. The bifidobacterial population in milk has been attributed to the existence of prebiotic oat in feeding systems. Since breast feeding protects the newborns against several infectious diseases, milk from sows fed with oat could improve the health of piglets.

**Abstract:**

The objectives of this study were to (i) investigate the impact of feeding oat on the population of bifidobacteria and (ii) evaluate their probiotic potential. In this study, we investigated the effects of supplementing sows’ gestation and lactation feed with 15% oat (prebiotic source) on the levels of probiotic population in milk. We found that dietary inclusion of oat during lactation and gestation resulted in increased levels of bifidobacteria compared to lactobacilli in sow milk. Furthermore bifidobacteria within the sow milk samples were further evaluated for probiotic potential based on aggregating properties, and acid- and bile-tolerance after exposure to hydrochloric acid (pH 2.5) and bile salts (0%, 0.25%, 0.50%, 1.0% and 2.0%). All isolates survived under the condition of low pH and bile 2.0%. Autoaggregation ability ranged from 17.5% to 73%. These isolates also showed antimicrobial activity against *E. coli* O157:H7. Together our results suggest that inclusion of oat in feeding systems could have the potential to improve the intestinal health of piglets by increasing the population of bifidobacteria.

## 1. Introduction

Maintaining healthy hogs and increasing production is important to the pork industry, consumers, and the economy. Many strategies are employed by the pork industry to increase production. One common strategy involves weaning piglets at approximately 21 days of age in order to allow for more piglet litters per sow each year [1]. While weaning piglets at early ages shortens the time-period when the sows are out of production, performance of the piglets can be negatively affected. Weaning piglets early is challenging because their digestive and immune systems are not yet fully mature [2]. Consequently, piglets are more susceptible to infections and gastrointestinal (GI) issues. One GI disease that piglets are prone to develop, particularly when they are weaned before four weeks of age, is post weaning diarrhea (PWD). PWD leads to anorexia, growth inhibition, and death of piglets and therefore is an important issue with economic consequences to the swine industry. To avoid post-weaning diarrhea, antibiotic growth promoters have been included in piglet diets; however, antibiotic growth promoters are potentially linked to the emergence of antibiotic resistant bacteria [3]. Therefore, alternatives to antibiotic growth promoters that maintain piglet health and boost immunity are being sought [4].

In swine, nutrients consumed during gestation and lactation has been shown to be key to the viability and health of offspring. Alexopoulos *et al.* [5] reported that supplementation of sow diets with probiotics during gestation and lactation has been shown to lead to increased body weights and reduced incidences of diarrhea in their offspring. To promote the health of weaned pigs and protect against PWD, it is important that the gut be colonized by beneficial bacteria and that strong intestinal/mucosal immunity develops. Beneficial bacteria, like bifidobacteria and lactobacilli can stimulate the immune system, inhibit the growth of pathogens, and reduce the incidence of diarrhea and constipation in pigs [6,7]. Research has shown that a mother’s diet has the potential to positively affect the overall health of her offspring and contribute to development of their offspring’s immune systems. For example, breastfed infants of mothers who consumed probiotics had fewer gastrointestinal issues such as diarrhea [8]. In this study, we sought to evaluate whether supplementation of feeding system with oat would lead to changes in the levels of probiotics in milk. The objectives of this study were to investigate the impact of feeding oat on the population of bifidobacteria and to evaluate their probiotic potential.

## 2. Experimental Section

### 2.1. Animals, Experimental Diet, and Milk Collection

The study consisted of two trials with 16 sows, each with sows of first to third parity. Sows were of Duroc, Landrace, Yorkshire, and Berkshire genetic backgrounds. The feeding treatment groups were: (1) control, and (2) ground whole oat (15%) (Table 1). The experimental feed was distributed during the last month of gestation and the first month post-partum. The sows had free access to feed and water. The diets were formulated to meet the protein (20%) and energy (3.415 Mcal DE/kg) requirements of the sow, and vitamins and minerals were supplemented according to the nutrient requirements of swine published by the U.S. National Research Council (NRC). To facilitate lactation prior to milk sample collection, 2 mL of oxytocin was given via IM injection to each sow each weeks for three weeks starting on day of farrowing. A total of 28 milk samples were collected from each sows. After the milk was collected, it was placed on ice and transported to the lab within 3 h of collection. For each weekly evaluation, samples were pooled so the end result was one 50 mL tube per diet condition. All animals were housed at the North Carolina A & T State University Swine Research Unit. Animal use and handling was approved by the Institutional Animal Care and Use Committee (IACUC) of North Carolina A & T State University.

**Table 1:** Feed formulation.

**Feed Ingredients**	**Gestation (% of Feed)**	
	**Control**	**Oat**
11102 NCDA Corn (Rolled)	80.3	65.38
Soybean Meal	13.8	13.8
Ground Whole Oat 15%	0	15
Corn (1/8) Micro-Flush	1	1
Limestone Fine	1.11	1.11
MON-CAL 21% P	2.05	2.05
Salt	0.5	0.5
Swine TM PX (KSU)	0.15	0.15
Swine Sow-Pig VIT	0.04	0.04
Poultry Fat	1	1
**Feed Ingredients**	**Lactation (% of Feed)**	
	**Control**	**Oat**
Corn NCDA	73	64.8
Soybean Meal 48%	17.6	10.8
Ground Whole Oat 15%	0	15
Corn (1/8) Micro-Flush	1	1
Limestone Fine	1.08	1.08
MON-CAL 21% P	2.38	2.38
L-Lysine 50%	0.25	0.25
Salt	0.5	0.5
Threonine	0.01	0.01
Swine TM Prmx (KSU)	0.15	0.15
Swine VTM Prmx	0.04	0.04
Poultry Fat	3.99	3.99

### 2.2. Enumeration of Bacterial Population

For the enumeration of *Lactobacillus* and bifidobacteria, each milk sample was plated onto Man-Rogosa-Sharpe (MRS) and Bifidobacterium Iodoacetate (BIM-25) agar media respectively. BIM-25 is a selective medium for isolation and enumeration of *Bifidobacterium* spp. [9]. Plates were incubated anaerobically at 37 °C for 48–72 h before colonies were counted. Since our interest was to isolate and characterize bifidobacteria, 66 colonies from the BIM-25 agar media were inoculated and incubated in Trypticase Phytone Yeast (TPY) broth for 24 h. Among these, 23 isolates were determined to grow well; 12 isolates that showed aggregation (determined by visual observation) were selected for the next phase of the study. In addition, two nonaggregating isolates were selected for evaluation of their other probiotic potential.

### 2.3. Identification of Bifidobacterium from Sow Milk

Initially, 14 selected colonies grown in BIM-25 agar were picked out, Gram’s stained and examined under the microscope for the cell morphology.

#### 2.3.1. Phenotypical Characterization

##### 2.3.1.1. Aggregation Assay

Autoaggregation was determined initially by visual inspection of broth cultures. Cultures were gently homogenized on a vortex for few seconds and left at room temperature. After 2 h, turbidity was measured by taking upper layer of bacterial suspension and autoaggregation ability was expressed as autoaggregation percentage using (AAg %) = 1 − (O.D. upper suspension/O.D. total suspension) × 100 [10].

##### 2.3.1.2. Antimicrobial Activity Assay

An agar spot test described by Awaisheh and Ibrahim [11], with slight modification, was used to determine the antimicrobial activity. Ten microliters of active culture from isolates was spotted on the surface of the MRS agar plate. A 100 μL of an overnight culture of indicator bacteria *E. coli* O157:H7 (944) was mixed with 7 mL of soft BHI agar (0.7%) and poured over the plate. The plates were incubated at 37 °C for 24 h and zones of inhibition (mm) were measured.

##### 2.3.1.3. Determination of Bile Tolerance

MRS broth supplemented with 0, 0.25, 0.5, 1.0, and 2.0 (w/v) ox-gall (Sigma Chemical Co., St. Louis, MO, USA.) were freshly prepared and sterilized by autoclaving at 121 °C for 15 min. A 0.1 mL of appropriately diluted overnight suspensions of each isolates was inoculated into the tubes containing different bile salt concentrations. Samples were incubated at 37 °C for 12 h, the bacterial growth was determined by measuring the optical density at 610 nm.

##### 2.3.1.4. Tolerance of Isolates at Low pH

A 0.1 mL of overnight suspensions of each isolates was inoculated into MRS broth adjusted to pH 2.5 using HCL. Bacterial populations were enumerated by plating the samples onto BIM-25 agar plates after incubation at 37 °C for 90 min. Survival of bacterial population was compared to the control sample (unacidified MRS broth) of pH 6.5. To determine whether these acid challenged isolates could be recovered, the isolates were further incubated at 37 °C for 24 h using 96-well microplate reader (BioTek Institute, Winooski, VT, USA).

##### 2.3.1.5. Antibiotic Susceptibility Test

Each bacterial isolate was inoculated (1%, v/v) in MRS broth supplemented with different concentrations of antibiotics (ampicillin at 3 and 4 mg/L, chloramphenicol at 4 and 6 mg/L, erythromycin at 1 and 2 mg/L, and gentamicin at 65 and 66 mg/L) and growth was examined after 24 h incubation at 37 °C using a 96-well microplate reader. Antibiotic concentrations were selected based on microbiological breakpoints values [12].

### 2.4. Data Preparation and Statistical Analysis

Graph Prism 5.0 was used to graph results and perform statistical analysis for bacterial population. One-way ANOVA with a Tukey’s multiple comparison post-test was used. Data used for bacterial characterization were analyzed using SAS version 9.2 (SAS Inst., Cary, NC, USA.). Comparison between groups was performed using a paired t-test or a one-way analysis of variance with post hoc analysis by Duncan multiple test. *P* < 0.05 was considered statistically significant.

## 3. Results

### 3.1. Population of Lactobacillus and Bifidobacterium Spp.

**Figure 1:**
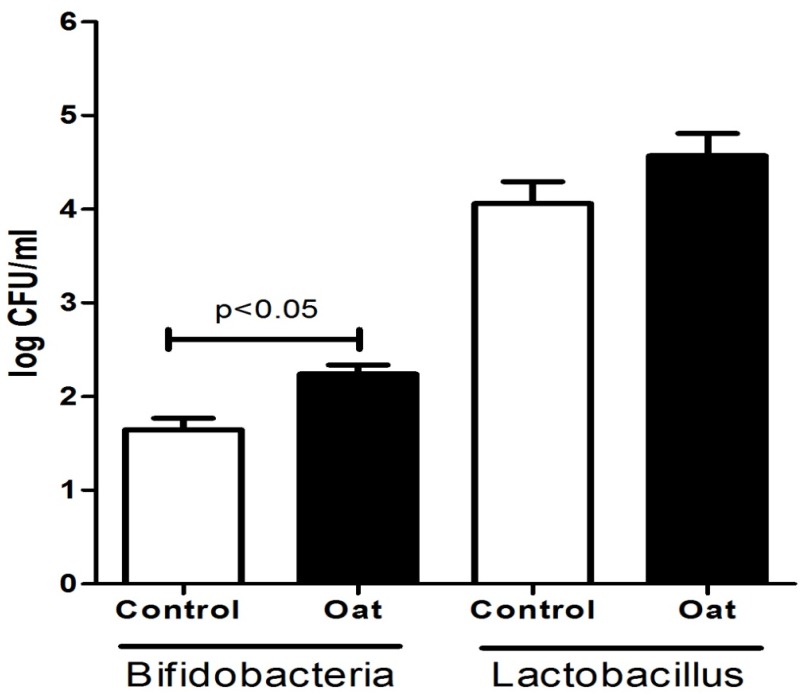
Comparison of bifidobacteria and *Lactobacillus* present in sow milk collected each week for three weeks post birth during trial 1 and trial 2. Each bar is an average of an n = 6.

Plate counts on milk samples collected from sows that were fed oat had significantly higher levels of bifidobacteria compared to (1) control group (*p* < 0.05) (Figure 1). In contrast, we did not observe significant changes in *Lactobacillus* levels between the diet groups.

### 3.2. Phenotypic Characterization

There are several studies on isolation and evaluation of probiotic potential especially lactobacilli from sow milk. To our knowledge this is the first study to isolate bifidobacteria from sow milk. In addition, our study showed significantly higher population of bifidobacteria compared to the control diet. Therefore, we sought to further characterize the probiotic potential of the bifidobacteria present in the sow milk. Bifidobacterial strains were isolated from sow milk using BIM-25 agar as a selective medium. Colonies from BIM-25 agar were examined under the microscope for morphological characterization. Gram-positive straight rod (branching and bifurcations) were selected as being the characteristics of bifidobacteria. They were thus identified as members of the genus bifidobacteria prior to further phenotypic characterization

#### 3.2.1. Autoaggregation and Antimicrobial Activity 

**Figure 2:**
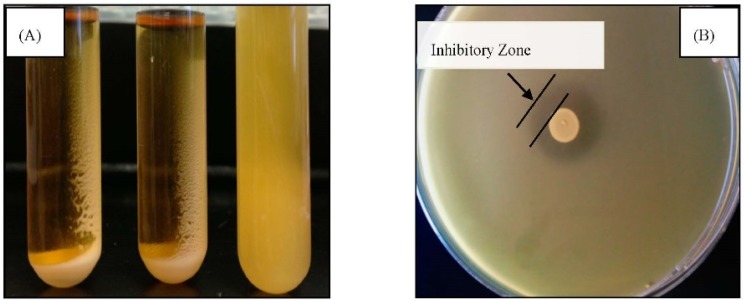
Macroscopic view of (**A**) autoaggregation and (**B**) antimicrobial property of tested isolate.

Based on the visual appearance of the cell suspensions, samples were selected for the autoaggregation assay. As shown in Figure 2A, autoaggregating isolates grown in MRS broth adhered around the surface of the tubes giving a clear supernatant (17.5% to 73% autoaggregation, Table 2). The non-aggregating strains showed turbid suspension. Based on our calculation, isolates were classified in different groups: high autoaggregation (>60%), medium autoaggregation (20%–60%), and low autoaggregation (<20%) [10]. Table 2 also shows the antimicrobial activity of isolates against indicator bacteria (*E. coli* O157:H7 strain 944). All isolates were able to inhibit indicator bacteria producing a clear zone of inhibition in the range of 6–11 mm in diameter (Figure 2B).

#### 3.2.2. Bile and Acid Tolerance

Table 3 shows the growth (absorbance readings at 610 nm) of 14 tested isolates in MRS broth containing bile concentration from 0%–2.0%. During 12 h of incubation at 37 °C, all the isolates survived well at a concentration level of bile of 2.0%. These results showed that all the isolates tolerated 2.0% bile with an absorbance readings ranging from 0.27–0.57 indicating resistance to 2.0% bile.

**Table 2:** Autoaggregation and antimicrobial activity of tested isolates.

Isolate No.	Autoaggregation (%)	Zone of Inhibition (mm)
1	Nonaggregating	10.0 ± 0.06
2	Nonaggregating	8.0 ± 0.07
3	17.5 ± 2.12	9.0 ± 0.04
4	25.5 ± 2.12	9.0 ± 0.06
5	68.0 ± 1.41	11.0 ± 0.04
6	45.5 ± 3.53	9.0 ± 0.08
7	62.0 ± 2.82	8.0 ± 0.06
8	73.0 ± 2.80	8.0 ± 0.04
9	66.0 ± 1.41	10.0 ± 0.05
10	60.5 ± 2.12	10.0 ± 0.03
11	51.0 ± 2.82	10.0 ± 0.04
12	63.5 ± 2.12	9.0 ± 0.05
13	64.0 ± 1.41	6.0 ± 0.06
14	25.0 ± 3.50	10.0 ± 0.03

The values are the averages of duplicate analysis ± S.D.

**Table 3:** Bile tolerance of the isolates observed by turbidity readings (O.D. 610 nm) after 12 h of incubation at 37 °C.

Isolate No.	Bile (%)				
	0%	0.25%	0.50%	1.00%	2.00%
1	0.98 ± 0.04	0.73 ± 0.04	0.54 ± 0.08	0.48±0.07	0.41 ± 0.11
2	0.95 ± 0.04	0.70 ± 0.08	0.47 ± 0.04	0.41±0.08	0.35 ± 0.12
3	0.93 ± 0.01	0.71 ± 0.08	0.51 ± 0.01	0.36±0.07	0.27 ± 0.08
4	0.82 ± 0.11	0.65 ± 0.01	0.46 ± 0.01	0.37±0.04	0.33 ± 0.10
5	0.93 ± 0.06	0.81 ± 0.03	0.68 ± 0.03	0.62±0.04	0.45 ± 0.11
6	1.02 ± 0.04	0.73 ± 0.04	0.72 ± 0.02	0.60±0.04	0.51 ± 0.05
7	1.01 ± 0.08	0.82 ± 0.04	0.74 ± 0.04	0.61±0.03	0.48 ± 0.04
8	0.96 ± 0.02	0.82 ± 0.03	0.73 ± 0.04	0.68±0.05	0.51±0.05
9	0.94 ± 0.06	0.74 ± 0.04	0.64 ± 0.04	0.65 ± 0.07	0.57 ± 0.04
10	0.89 ± 0.07	0.69 ± 0.04	0.53 ± 0.04	0.48 ± 0.01	0.43 ± 0.02
11	0.90 ± 0.05	0.72 ± 0.01	0.52 ± 0.05	0.43 ± 0.04	0.38 ± 0.04
12	0.85 ± 0.05	0.69 ± 0.01	0.54 ± 0.03	0.42 ± 0.04	0.36 ± 0.04
13	1.03 ± 0.18	0.95 ± 0.04	0.58 ± 0.02	0.53 ± 0.03	0.34 ± 0.04
14	1.01± 0.11	0.71 ± 0.04	0.56 ± 0.02	0.47 ± 0.02	0.41 ± 0.04
Average	0.94 ± 0.07 ^a^	0.75 ± 0.03 ^b^	0.59 ± 0.04 ^c^	0.51 ± 0.04 ^d^	0.41 ± 0.03 ^e^

The values are the averages of duplicate analysis ± S.D. Average values with the different superscript letters indicate significant difference (*p* < 0.05).

**Figure 3:**
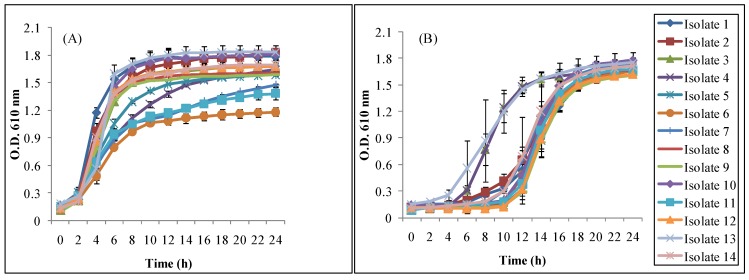
Growth of isolates in MRS broth after 90 min of incubation at pH (**A**) 6.5 and (**B**) 2.5.

Table 4 shows the population of isolates before and after 90 min of incubation at pH 6.5 and 2.5 at 37 °C. All the isolates were able to survive after exposure to pH 2.5 for 90 min. The initial population ranged from 6.48–9.56 Log CFU/mL. In the control sample (pH 6.5), the population reached to 7.21–9.77 Log CFU mL/mL after 90 min of incubation. Significant differences (*p* < 0.05) were observed when mean populations were compared using a paired t-test at pH 6.5 and 2.5 at 0 and 90 min. However, all the isolates were able to survive conditions mimicking the gastro intestinal environment. The isolate population ranged from 2.35 to 4.93 Log CFU/mL. Since stress recovery is an important criteria to determine the effectiveness of probiotic, we further determined whether these acid-stressed isolates could recover (grow). Figure 3 shows the recovery and growth of all the tested isolates at 37 °C for 24 h. After 8–10 h, all the isolates showed full recovery and continued to grow (Figure 3B). These isolates continued growing further and reached the maximum absorbance of 1.61 to 1.78 (O.D. 610 nm) similar to the reference isolates (Figure 3A) that were grown at pH 6.5 under the same condition.

**Table 4:** Population (Log CFU/mL) of isolates before and after 90 min of incubation at pH 6.5 or 2.5 at 37 °C.

Isolate No.	pH 6.5		pH 2.5	
	0 min	90 min	0 min	90 min
1	7.32 ± 0.13	7.72 ± 0.12	7.31 ± 0.02	2.83 ± 0.03
2	7.07 ± 0.06	7.74 ± 0.04	6.84 ± 0.06	2.63 ± 0.14
3	7.14 ± 0.03	7.41 ± 0.02	6.48 ± 0.07	2.35 ± 0.15
4	6.63 ± 0.04	7.21 ± 0.01	6.48 ± 0.02	2.65 ± 0.14
5	9.37 ± 0.24	9.41 ± 0.10	9.24 ± 0.05	3.87 ± 0.04
6	9.56 ± 0.08	9.77 ± 0.02	9.42 ± 0.04	3.31 ± 0.19
7	9.47 ± 0.11	9.56 ± 0.17	9.41 ± 0.09	2.70 ± 0.15
8	9.49 ± 0.04	9.51 ± 0.05	9.27 ± 0.10	3.30 ± 0.04
9	8.46 ± 0.02	8.73 ± 0.07	8.39 ± 0.10	2.64 ± 0.09
10	8.36 ± 0.04	8.62 ± 0.19	8.17 ± 0.05	2.67 ± 0.16
11	8.74 ± 0.05	8.80 ± 0.03	8.26 ± 0.06	3.34 ± 0.06
12	8.33 ± 0.10	8.88 ± 0.19	8.08 ± 0.13	4.06 ± 0.02
13	9.01 ± 0.01	9.18 ± 0.07	8.11 ± 0.04	4.93 ± 0.03
14	8.18 ± 0.02	9.00 ± 0.08	7.93 ± 0.10	4.01 ± 0.07
Average	8.36 ± 0.07	8.68 ± 0.08	8.10 ± 0.07	3.23 ± 0.09

The values are the averages of duplicate analysis ± S.D.

#### 3.2.3. Antibiotic Susceptibility

Table 5 shows the antibiotic susceptibility test of 14 isolates to four different antibiotics at different concentrations. Bacterial strains were considered resistant when they showed MIC values higher than the MIC breakpoints established by the European Food Safety Authority [12]. All the tested isolates were sensitive to ampicillin and chloramphenicol and resistant to gentamicin at tested concentration. However, with antibiotic erythromycin, isolates 1, 8, 12, 13 and 14 showed sensitivity while the rest of the isolates were resistant, according to the breakpoints set by EFSA, indicating that these isolates could be strain-dependent.

**Table 5:** Antibiotic susceptibility of different isolates.

Antibiotics (mg/L)								
Isolate No.	Ampicillin		Chloramphenicol		Erythromycin		Gentamicin	
	4	5	5	6	1	2	65	66
**1**	S	S	S	S	S	S	R	R
**2**	S	S	S	S	R	R	R	R
**3**	S	S	S	S	R	R	R	R
**4**	S	S	S	S	R	R	R	R
**5**	S	S	S	S	R	R	R	R
**6**	S	S	S	S	R	R	R	R
**7**	S	S	S	S	R	R	R	R
**8**	S	S	S	S	S	S	R	R
**9**	S	S	S	S	R	R	R	R
**10**	S	S	S	S	R	R	R	R
**11**	S	S	S	S	R	R	R	R
**12**	S	S	S	S	S	S	R	R
**13**	S	S	S	S	S	S	R	R
**14**	S	S	S	S	S	S	R	R

Antibiotic concentrations were selected based on microbiological breakpoints values (mg/L) for *Bifidobacterium* spp. [12]. S-Sensitive, R-Resistant.

## 4. Discussion

Hog production is an important industry to the US and worldwide market. Post weaning diarrhea is responsible for major economic losses due to mortality, morbidity, decreased growth rate, and cost of medication [13]. PWD is a multifactorial disease that can be caused by many different agents. Several pathogens that cause diarrhea include *Lawsonia* spp., *Clostridium* spp., *Brachyspira* spp., *Campylobacter* spp., *Salmonella* ssp., coronaviruses, and transmissive gastroenteritis viruses [4,14]; however, in the swine industry, the most common cause of post-weaning diarrhea is enterotoxigenic *Escherichia coli* [15].

To avoid post-weaning diarrhea, antibiotic growth promoters have been included in piglet diets. However, antibiotic growth promoters are known to suppress beneficial organisms in the gut and, because of a possible link to the emergence of antibiotic resistant bacteria, there is a push to phase out or eliminate their use in swine production [16]. Intestinal microflora is a key contributor to intestinal health. Among probiotic genera, bifidobacteria are one of the predominant bacterial groups existing in the animal and human intestine [17] and are believed to play a beneficial role in maintaining the health of the host.

Our study showed that the inclusion of oat diet increased the bifidobacterial population more than lactobacilli. The possible explanation for this could be that oat contains oligosaccharides that function as prebiotic for the bacterial growth including bifidobacteria [18]. Tuohy *et al.* [19] reported that bifidobacteria as specialized oligosaccharides-degrading bacteria. These bacteria have a broad range of glycolytic enzymes and carbohydrate uptake channels with high affinity for prebiotic oligosaccharides and their composite sugars. These prebiotics could have served as a growth-promoting factor for bifidobacteria [19]. This is further confirmed by Hinz *et al.* [20], who also reported that bifidobacteria play an important role in degradation of oligosaccharides. Consistent with our findings, a study conducted by Connolly *et al.* [21] showed that oat flakes increase bifidobacteria populations *in vitro*, which also suggest that oats have prebiotic properties. Additionally, using culture, isolation, sequencing, and fingerprinting methods, Jost *et al.* [22] demonstrated that the same probiotic bacteria are found in the feces of mothers and offspring as well as in mother’s milk, suggesting that the probiotic bacteria travel from mother’s gut into milk. Therefore, in our study, one can speculate that the diet containing oat led to increased bifidobacteria in the intestine of the sows and that these bacteria travelled to the milk.

The principle criterion for selection of a good probiotic is to overcome physiological barriers through the stomach (acid pH) and small intestine (bile salt) in order to arrive in the large intestine in a viable physiological state [23]. In addition, these probiotics should adhere to the intestinal mucosa, possess antimicrobial properties, and should not have transmissible antibiotic resistance genes [24,25]. Acid and Bile tolerance are considered to be an important characteristic of probiotic strains. This tolerance supports survival and growth of probiotics and thus allows probiotics to exert their beneficial effects in the gastrointestinal tract [24]. Yun *et al.* [26] reported the survivability of *Lactobacillu*s strains isolated from pig feces at 1% (w/v) bile, which is the condition found in the stomach and intestine. All the isolates tested in our study were able to survive at 2.0% (w/v) bile concentration. However, bile salt tolerance usually varied among the strains of bifidobacteria [27].

It has been reported that probiotic strains, and particularly bifidobacterial strains, have lower acid tolerance system, with the exception of *B. animalis* subsp. *Lactic* [28]. However, probiotic strains may have adaptive capabilities to survive in acidic conditions, allowing the strains to improve recovery and to continue growth. Despite having a slightly low bacterial count (Log CFU/mL), growth of isolates were not affected following 90 min exposure to pH 2.5 when further grown in pH 6.5 MRS broth. The basic requirement for probiotics to exert expected positive effects is to be viable. Similarly, all the isolates were capable of surviving and growing at pH 2.5 and 1% (w/v) bile salt, which are the conditions, found in the stomach and intestine. All the tested isolates survived well in low pH (2.5) and showed a similar growth trend. Acid tolerance response (ATR) may have developed in these isolates after the exposure of cells to low acid (pH 2.5), which might account for the isolates’ good growth after the recovery period [29].

Aggregation properties of probiotic bacteria indicate that they can adhere to mucosal surfaces that will have prolonged survival in the body of the host. This property of probiotics bodes well for the use of probiotics and could be promising candidates for use in animal feed or to develop functional foods [30]. Ibrahim *et al.* [10] studied several bifidobacterial strains and classified them into autoaggregation sensitive (≥70%), moderate (20%–60%), and resistant groups (<20%) similar to our classification of high, medium and low autoaggregation behavior. In the present study, all the isolates also showed a clear zone of inhibition against *E. coli* O157:H7. From these observed zones, it is apparent that these isolates possess antimicrobial properties.

The determination of antibiotic susceptibility of intestinal microorganisms is another important criterion for selecting an organism as a probiotic. Agaliya and Jeevaratnam [31] reported the resistivity of *L. plantarum* isolated from fermented olives towards gentamicin (10 μg/disc) whereas strains were sensitive to ampicillin (10 μg), chloramphenicol (30 μg), and erythromycin (15 μg). Argyri *et al.* [23] also reported the resistivity of various lactic acid bacteria strains to ampicillin, chloramphenicol, erythromycin, and gentamicin when concentrations were used above MIC values set by EFSA. High resistance rates were observed with low-level kanamycin (30 μg) and gentamicin (15 μg) for 100% of the tested strains of *Bifidobacterium* spp., similar to our results. However, tested strains were sensitive to high-level of kanamycin (1000 μg) or gentamicin (500 μg,) using the disc diffusion method [32]. Similarly in another study, *Bifidobacterium* spp. isolated from dairy and pharmaceutical products were resistant to gentamicin (MIC = 0.5 μg/mL), and susceptible to chloramphenicol (MIC = 2 μg/mL), erythromycin (MIC = 0.5 μg/mL), and ampicillin, (MIC = 4, μg/mL) [33]. In cases of co-administration with antibiotics to prevent and treat intestinal disorders, probiotics should be resistant to certain antibiotics in order to survive in the gastrointestinal tract [34]. However, the use of antibiotic-resistant probiotic strains is still a controversial subject as antibiotic-resistant genes could transfer to the other bacteria present in the host gastrointestinal tract [35,36]. In addition, if such probiotic bacteria are added to different feed products, they could contribute to a potential source for the spread of antibiotic-resistance genes [33]. Therefore, it is still a debatable subject whether one should select antibiotic-resistant or sensitive strains. Thus, more work is needed to address the potential impact of antibiotic-resistant bacteria to the host.

## 5. Conclusions

In this study, we isolated potential probiotic bifidobacteria from sow milk. Our study suggests that inclusion of oat in feeding could have the potential to improve the intestinal health of piglets by increasing the population of bifidobacteria. However, further research on long term supplementation of oats in feeding systems is needed before any recommendation about oats' beneficial effects on pig health can be made. It is equally important to identify these isolates up to species and strain level before being used in any products. Further studies on the antimicrobial activity of these isolates against other pathogens, adhesion to intestinal epithelial cells, characterization of bacterial cell wall composition, and cell surface hydrophobicity is also required.
